# Enhanced G2/M Arrest, Caspase Related Apoptosis and Reduced E-Cadherin Dependent Intercellular Adhesion by Trabectedin in Prostate Cancer Stem Cells

**DOI:** 10.1371/journal.pone.0141090

**Published:** 2015-10-20

**Authors:** Eda Acikgoz, Ummu Guven, Fahriye Duzagac, Ruchan Uslu, Mikail Kara, Burak Cem Soner, Gulperi Oktem

**Affiliations:** 1 Department of Histology and Embryology, Ege University Faculty of Medicine, Bornova, Izmir, Turkey; 2 Department of Stem Cell, Ege University Health Science Institute, Bornova, Izmir, Turkey; 3 Department of Medical Oncology, Ege University Faculty of Medicine, Bornova, Izmir, Turkey; 4 Department of Histology and Embryology, Yuzuncu Yil University Faculty of Medicine, Van, Turkey; 5 Department of Medical Pharmacology, Necmettin Erbakan University, Meram Faculty of Medicine, Konya, Turkey; Taipei Medical University, TAIWAN

## Abstract

Trabectedin (Yondelis, ET-743) is a marine-derived tetrahydroisoquinoline alkaloid. It is originally derived from the Caribbean marine tunicate *Ecteinascidia turbinata* and currently produced synthetically. Trabectedin is active against a variety of tumor cell lines growing in culture. The present study focused on the effect of trabectedin in cell proliferation, cell cycle progression, apoptosis and spheroid formation in prostate cancer stem cells (CSCs). Cluster of differentiation (CD) 133^+high^/CD44^+high^ prostate CSCs were isolated from the DU145 and PC-3 human prostate cancer cell line through flow cytometry. We studied the growth-inhibitory effects of trabectedin and its molecular mechanisms on human prostate CSCs and non-CSCs. DU-145 and PC-3 CSCs were treated with 0.1, 1, 10 and 100 nM trabectedin for 24, 48 and 72 h and the growth inhibition rates were examined using the sphere-forming assay. Annexin-V assay and immunofluorescence analyses were performed for the detection of the cell death. Concentration-dependent effects of trabectedin on the cell cycle were also evaluated. The cells were exposed to the different doses of trabectedin for 24, 48 and 72 h to evaluate the effect of trabectedin on the number and diameter of spheroids. According to the results, trabectedin induced cytotoxicity and apoptosis at the IC_50_ dose, resulting in a significant increase expression of caspase-3, caspase-8, caspase-9, p53 and decrease expression of bcl-2 in dose-dependent manner. Cell cycle analyses revealed that trabectedin induces dose-dependent G2/M-phase cell cycle arrest, particularly at high-dose treatments. Three-dimensional culture studies showed that trabectedin reduced the number and diameter of spheroids of DU145 and PC3 CSCs. Furthermore, we have found that trabectedin disrupted cell-cell interactions via E-cadherin in prostasphere of DU-145 and PC-3 CSCs. Our results showed that trabectedin inhibits cellular proliferation and accelerates apoptotic events in prostate CSCs; and may be a potential effective therapeutic agent against prostate cancer.

## Introduction

The cancer stem cells (CSCs) hypothesis states that tumors contain only a small subpopulation of cells with a potential of self-renewal and differentiation. CSCs are thought to be responsible for tumor initiation and maintenance of tumor growth and cell survival after chemotherapy due to their resistance to conventional anticancer therapies [1]. During early tumor development, CSCs may undergo a symmetrical self-renewing cell division into two identical daughter CSCs but also generate bulk populations of non-CSCs by asymmetrical cell division [2]. The majority of cells in bulk tumors have limited tumorigenic and metastatic potential when compared to CSCs. For a more effective treatment of cancer, it may be necessary to target both CSCs and non-CSC populations.

CSCs have been previously isolated using CSC-specific cell surface markers such as CD44, CD133, CD24, α2β1 integrin and aldehyde dehydrogenase1. CD133 and CD44 are the most commonly used cell*-*surface markers for identification of CSCs. CD133 is a member of pentaspan transmembrane glycoproteins. It was first described on hematopoietic and neural stem/ progenitor cells and is also a marker for CSCs in many solid tumors [3, 4]. CD44 is a ubiquitous multi-structural and multi-functional cell surface glycoprotein. It is involved in cell adhesion, migration and metastasis of a variety of tumor cells and stemness regulation of CSCs [5]. It has been previously shown that a CD44^+^/α2β1^high^/CD133^+^ phenotype represent the candidate prostate cancer tumorigenic cells [6]. Therefore, CD133^+^/CD44^+^ cells could be potential targets of antitumor therapy in the future.

Conventional monolayer two-dimensional (2D) cell culture studies have been successful in explaining the behaviour of CSCs. On the other hand, in vitro three-dimensional (3D) cancer model mimics the features of the in vivo environment and therefore provides a better opportunity to understand crucial cancer stem cell mechanisms and to develop new clinical therapeutic applications [7]. In vitro, CSCs tend to spontaneously to form three-dimensional cellular aggregates, called spheroids which represent the differentiation properties of CSCs and are used for contributing to tumor generation, progression and chemotherapy resistance in several studies. It has been shown that E-cadherin is the major adhesion molecule mediating tight cell-cell interaction and has been correlated with a compact spheroid formation in prostate cancer cell lines [8, 9].

Trabectedin is a marine tetrahydroisoquinoline alkaloid. Trabectedin has been isolated from the Caribbean marine tunicate *Ecteinascidia turbinata* and is currently produced synthetically [10]. Trabectedin has a potent cytotoxic activity against a variety of tumor types in several solid tumours *in vitro* and *in vivo*. It is undergoing phase I and II clinical trials in Europe and the United States with promising a anticancer drug for the treatment of a range of tumours [11]. Nevertheless anticancer activity and mechanism of action remains unclear. It has been shown that trabectedin binds to the the N2 position of guanines in the minor groove of DNA, bending DNA towards the major groove and interferes with several transcription factors and DNA repair pathways [11]. It is also known that trabectedin induces DNA damage. This alters the normal function of DNA repair and transcription processes, resulting with an arrest of proliferation, differentiation and cell death. [11, 12, 13]. The anti-proliferative activity of trabectedin is dose dependent: at low concentrations (1–10 ng/ml), trabectedin produces cell cycle perturbations with a decreased rate of S-phase progression and accumulation of cells in G2 phase. At higher concentrations (10–100 ng/ml), transcription-independent process leads to apoptosis via activating different signal transduction pathways involving mitochondrial cytochrome c release, JNK and caspase- 3 activation [14]. The cytostatic and pro-apoptotic activities of trabectedin result from the activation of the intrinsic and/or extrinsic apoptotic routes. The intrinsic pathway is characterized by mitochondrial outer membrane permeabilization and release of cytochrome-c into the cytoplasm; a process regulated by the Bcl-2 family of proteins. Previous work suggests that trabectedin triggers cytochrome c release from mitochondria that is normally inhibited by Bcl-2 overexpression [14].

Recently, emerging evidences have shown that CSCs play critical roles in the development of drug resistance, metastasis and recurrence. Thus, it is important to investigate and find new drugs which will selectively and effectively target and kill CSCs. The current study aimed to investigate the effects of trabectedin in CD133^+high^/CD44^+high^ prostate CSCs in 2D and 3D culture system.

## Materials and Methods

### Cell culture conditions and reagents

Human hormone- and drug-resistant prostate cancer cell lines, PC-3 and DU145 were purchsed from American Type Culture Collection (Manasas, VA, USA) and were grown in RPMI 1640 (*Lonza*, *Basel*, *Switzerland*) culture medium containing 10% heat-inactivated fetal bovine serum (Gibco, Invitrogen Life Technologies, Paisley, UK), 1% penicillin and streptomycin (Sigma-Aldrich, St Louis, MO, USA). Cells were cultured in 25 cm^2^ polystyrene flasks (Corning Life Sciences, UK) and maintained in an incubator at 37°C in a humidified atmosphere in the present of 5% CO_2_. Growth and morphology were checked microscopically daily to ensure cell health. Cells were split passaged when they had reached approximately an 80% confluency. Cells in semiconfluent flasks were harvested using 0.05% trypsin (Sigma-Aldrich) and centrifuged (Nuve NF200; Laboratory and Sterilization Technology, Ankara, Turkey) after the addition of RPMI 1640 for trypsin inactivation. After centrifugation they were resuspended in culture medium. Trabectedin was provided by PharmaMar (Madrid, Spain) and was prepared as a 2 mM stock solution in dimethyl sulphoxide (DMSO). The DMSO concentration in the assay did not exceed 0.1% and was not cytotoxic to the tumor cells. Antibodies used were anti-caspase-3 (1:100 diluted; 3510-100, BioVision, Inc., Milpitas, CA, USA), anti-caspase-8 (1:100 diluted; 250576, Abbiotec, USA), anti-caspase-9 (1:100 diluted; SantaCruz Biotechnology, USA, sc-17784), anti-p53 (1:100 diluted; 3036R-100, BioVision, Inc.), anti-bcl2 (1:100 diluted; SantaCruz Biotechnology, USA, sc-135757), anti-E-cadherin (1:100 diluted; Bios, USA, bs-1519R), goat anti-rabbit immunoglobulin G-fluorescein isothiocyanate (FITC) (1:100 diluted; sc-2012, Santa Cruz Biotechnology, Inc., Santa Cruz, CA, USA) and chicken anti-rabbit immunoglobulin Alexa fluor ^®^ 594 (1:100 diluted; İnvitrogen, USA, A21442).

### Fluorescence-activated cell sorting (FACS)

Prior to harvesting, the cell lines were grown until an 80% confluency. For FACS (FACSAria; BD Biosciences, San Jose, CA, USA), the cells were detached using non-enzymatic cell dissociation solution (Sigma-Aldrich) and resuspended in Dulbecco's phosphate-buffered saline (DPBS, Invitrogen,USA). Approximately 5x10^4^ cells were incubated with an antibody (diluted 1:100 in FACS wash with 0.5% bovine serum albumin; 2 mM NaN3 and 5 mM EDTA) for 15 min at 4°C. An isotype and concentration-matched phycoerythrin (PE)-labeled control antibody (Miltenyi Biotec Ltd., Woking, Surrey, UK) was used and the samples were labeled with PE-labeled CD133/1 (clone AC133/1; Miltenyi Biotec Ltd.) and FITC-labeled CD44 (clone G44-26; BD Biosciences). After 3–5 minutes, the cells were washed and subsequently re-suspended. The cells were sorted to be CD 133^high^/ CD44^high^ population (sorting cells) and non-sorting counterparts using a FACSAria flow cytometer, with post-sort analysis performed to confirm population purity. Sorted cell populations were cultured in two different settings, monolayer 2D culture or 3D multicellular tumor spheroid.

### Cell viability analyses

Viability of the cells following treatment was determined using the Muse™ Count and Viability kit (Muse™Cell Analyzer; Millipore, Billerica, MA, USA) according to the manufacturer’s instructions. Briefly, cells were seeded in triplicate in 6-well plates at a density of 1×10^4^cells/well. After 24 h incubation, cells were exposed to increasing concentrations of trabectedin (0.1, 1, 10, 100 nM). Then, plates were incubated at 37^°^C in a 5% CO_2_ incubator for 24, 48, and 72 h. After incubation, all cells were collected and diluted with phosphate buffered saline (PBS). 50 μl of cell suspension was then added into 450 μl MUSE Count and Viability reagent (10x dilution), incubated for 5 min at room temperature, and analyzed using the MUSE Cell Analyzer. Data were presented as proportional viability (%) by comparing the trabectedin treated group with the untreated cells, the viability of which is assumed to be 100%.

### Cell death analyses

The apoptotic cell distribution was determined using the MUSE Annexin V & Dead Cell Kit (Merck KGaA) according to the manufacturer’s instructions. Briefly, after treatment with trabectedin, all cells were collected and diluted with PBS containing 1% bovine serum albumin (BSA) as a dilution buffer to a concentration of 5x10^5^ cells/ml. 100 μL of Annexin V/ dead reagent and 100 μL of a single cell suspension were mixed in a microtube and in the dark for 20 min at room temperature. Cells were then analyzed using the Muse cell analyzer (Merck Milipore). The apoptotic ratio was determined by the identification of four populations: (i) nonapoptotic cells, not undergoing detectable apoptosis: Annexin V (−) and 7-AAD (−); (ii) early apoptotic cells, Annexin V (+) and 7-AAD (−); (iii) late apoptotic cells, Annexin V (+) and 7-AAD (+); (iv) cells that have died through nonapoptotic pathway: Annexin V (−) and 7-AAD (+). The samples were determined by the Muse Cell Analyzer (Merck Millipore).

### Cell cycle analyses

Cell cycle analyses were performed using a Muse™ cell cycle kit from Millipore according to the manufacturer’s instructions. Briefly, cells were grown in 6-well plates treated with various concentrations of trabectedin (0.1, 1, 10, 100 nM); then harvested by trypsinization and washed twice with PBS. The cells were fixed with 1ml of 70% cold ethanol at -20°C for 5 h and treated with 200 μl of Muse cell cycle reagent and incubated for 30 min at room temperature in the dark. The percentage of cells in G0/G1, S and G2/M phases was then calculated using a Muse cell analyzer (Millipore, USA).

### Sphere formation and colony formation assays

The spheroid formation potential of CD133^high^/CD44^high^ human prostate CSCs was evaluated in 3D non-adherent culture condition. Initially the CD133^high^/CD44^high^ human prostate CSCs were grown as a monolayer after which they were counted, re-suspended and plated with 1x10^4^ cells per well in a 6-well plate pre-coated with a thin layer of 3% Noble agar (w/v) (Difco Laboratories, Inc.; BD Diagnostic Systems, Detroit MI, USA) in RPMI 1640 containing 10% FBS and incubated at 37°C in a humidified atmosphere of 5% CO_2_. Cell culture media was replaced every 2-3 days with fresh medium to remove cellular debris and the spheroids that were not well-formed. After the beginning of the multicellular tumor spheroid formation, trabectedin was added at various concentrations of trabectedin (0.1, 1, 10, 100 nM) and incubated for 24, 48 and 72 h. The number and diameter of colonies within each well was photographed and counted every day under the microscope (Olympus BX-51; Olympus, Hamburg, Germany) and the images of the representative fields were captured. Each sample was analysed in triplicate and all the experiments were performed three times.

### Immunofluorescence staining

Following treatment with IC_50_ dose of trabectedin, cells were placed on lysine coated coverslips, fixed in 4% paraformaldehyde for 15 min. Subsequently, the cells were permeabilized with 0.1% Triton X-100 for 10 min at room temperature, washed three times with PBS, blocked with PBS containing 5% bovine serum albumin for 1 h and incubated with primary antibodies against caspase-3, caspase-8, caspase-9 p53, bcl-2 and e-cadherin for overnight at 4°C. Then, the cells were treated with the secondary antibody for 1 h at room temperature in a humidified chamber. The immunostained cells were mounted in mounting medium containing DAPI and were visualized by a fluorescence microscope equipped with a camera (Olympus BX-51 and the Olympus C-5050 digital test).

### Statistical analysis

Experiments were carried out in triplicate. Statistical analysis was performed by using one-way analysis of variance, followed by Tukey's or Dunett's post hoc test. p<0.05 was considered to indicate a statistically significant difference.

## Results

### Purity and sorting rates of CD 133^high^/ CD44^high^ sorted and non-sorted subpopulation

**Fig 1:**
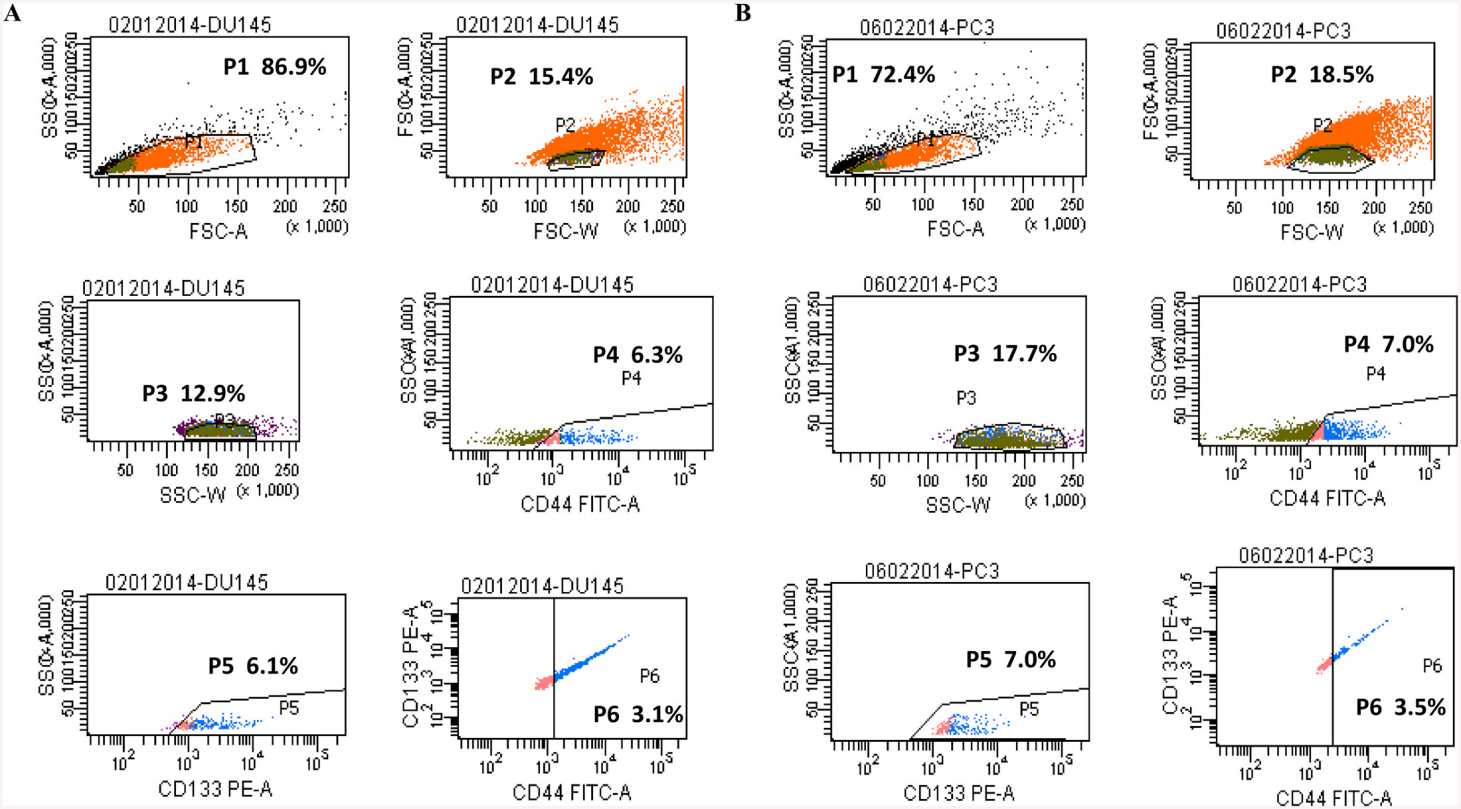
CD133^high^/ CD44^high^ prostate cancer stem cells isolated with the FACS-Aria cell sorter. (A) DU-145 CSCs were isolated from DU-145 human prostate cell line. (B) PC-3 CSCs were isolated from DU-145 human prostate cell line. CD133^high^/ CD44^high^ populations presented in P6. CD, cluster of differentiation; FACS, fluorescence-activated cell sorting.

DU145 and PC-3 human prostate cancer cells were sorted for CD133 and CD44 surface expression with FACS (Fig 1A and 1B). Results showed that the rates of DU-145 CSCs and non-CSCs were 3.2 ±5.4% (Fig 1A) and 96.8 ±5.4% (Fig 1B), respectively. In PC-3 cells, rates were 3.9 ±5.4 for sorting cells and 96.1 ±5.4 for non-sorting cells. A post-sort analysis was performed to determine the purity of the sorted cell populations; which was found to be >85%.

### Increasing cytotoxicity of CD133^high^/CD44^high^ prostate CSCs with trabectedin

To determine the effect of trabectedin on the viability of human prostate epithelial cells (RWPE-1), prostate cancer cells (DU-145 and PC-3), prostate CSCs (DU-145 and PC-3 CSCs) and bulk population (DU-145 non-CSCs and PC-3 non-CSCs) were exposed to increasing concentrations of trabectedin (0.1–100 nM) for 24, 48 and 72 h, and the percentage of viable cells in the samples were determined by cell viability assay.

**Fig 2:**
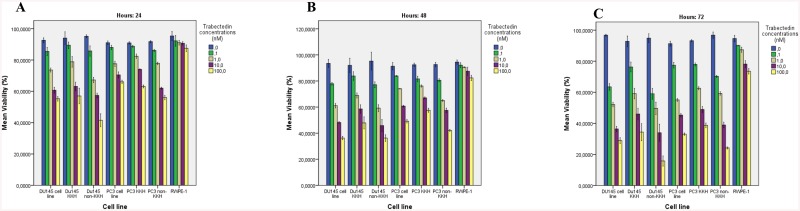
Effect of trabectedin on the cell viability after 24, 48 and 72 h following trabectedin treatment. Cytotoxicity was determined by the Muse™ cell analyzer. The results are expressed as the mean of 3 different experiments (±SD) (p< 0.001 compared to untreated control).

Trabectedin reduced cell viability in DU145 human prostate cell line, DU-145 CSCs and DU-145 non-CSCs in a time- and concentration- dependent manner (Fig 2). After 24 h treatment, The half maximal inhibitory concentration (IC_50_) values of trabectedin was found to be 100 nM in DU-145 non-CSCs; whereas IC_50_ value of trabectedin in DU-145 cell line and CSCs could not be obtained (Fig 2A). After 48 h treatment, the IC_50_ values of trabectedin were found to be 10 nM, 100 nM and 9,2 nM respectively in the DU-145 cell line, DU-145 CSCs and DU-145 non-CSCs (Fig 2B). After 72 h treatment, the IC_50_ values of trabectedin were found to be 1 nM, 9.3 nM and 1 nM respectively in the DU-145 cell line, DU-145 CSCs and DU-145 non-CSCs (Fig 2C).

Trabectedin decreased the cell viability in PC-3 human prostate cell line, PC-3 CSCs and PC-3 non-CSCs in a time- and concentration- dependent manner (Fig 2). IC_50_ value of trabectedin could not be obtained in all three group for 24 h. After 48 h treatment, the IC_50_ values of trabectedin were found to be 100 nM in the PC-3 cell line and PC-3 non-CSCs; whereas IC_50_ value of trabectedin in PC-3 CSCs could not be obtained (Fig 2B). After 72 h treatment, the IC_50_ values of trabectedin were found to be 9 nM, 10 nM and 8 nM respectively in PC-3 cell line, PC-3 CSCs and PC-3 non-CSCs (Fig 2C). Although, trabectedin reduced the viability of CSCs and non-CSCs in a concentration-dependent manner, the viability of RWPE-1 cells was significantly higher than prostate cancer cells after exposure to trabectedin, indicating that prostate cancer stem cells were more sensitive to trabectedin than normal prostate epithelial RWPE-1 cells.

### Trabectedin induced apoptotic cell death both CSCs and non-CSCs

**Fig 3:**
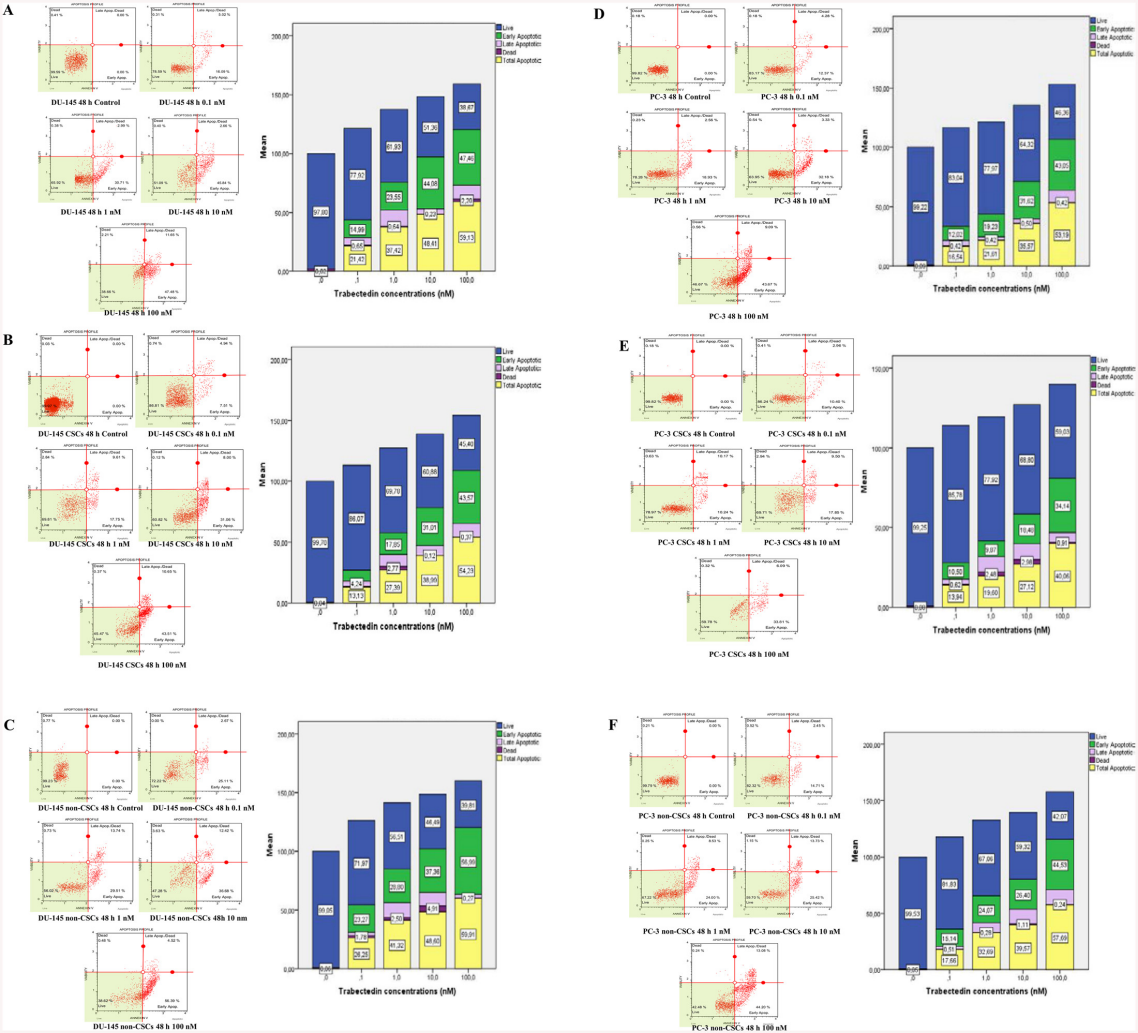
Trabectedin induced apoptosis in a dose-dependent manner as measured by the Muse™ Annexin V and Dead Cell assay. (A) DU-145 cell line, (B) DU-145 CSCs, (C) DU-145 non-CSCs, (D) PC-3 cell line, (E) PC-3 CSCs, (F) PC-3 non-CSCs. Cells were treated with 0.1, 1, 10 and 100 nM trabectedin for 48 h. After incubation time cells were collected and the phosphatydilserine externalization was evaluated using Annexin V protocol as describe. Based on Annexin V reactivity and the intensity of the 7-AAD fluorescence, cells can be classified into four categories: dead, live, early apoptosis and late apoptosis/dead. Trabectedin was shown to induce apoptotic cell death in the cancer stem cells mainly with the increase in early apoptosis (green), cells and the apparent decreased in the percentage of live cells. Trabectedin also significantly induced the total apoptotic cells (yellow) in a dose-dependent manner. Data represent the mean ± SEM of three different experiments. Statistical significance was determined with one-way analysis of variance, followed by Tukey's or Dunett's post hoc test. p<0.05 was considered to indicate a statistically significant difference.

To examine whether cells undergo apoptosis, untreated or trabectedin-treated DU-145 cell line, DU-145 CSCs, DU-145 non-CSCs, PC-3 cell line, PC-3 CSCs, PC-3 non-CSCs were exposed to increasing concentrations of trabectedin and evaluated by the Muse™ Annexin V and Dead Cell assay (Fig 3). Annexin V and Dead Cell analysis of cells can distinguish cells into four groups, namely viable (Annexin V (−) and 7-AAD (−), early apoptosis Annexin V (+) and 7-AAD (−), late apoptosis, Annexin V (+) and 7-AAD (+) and necrotic Annexin V (−) and 7-AAD (+). Trabectedin significantly induced apoptosis in all groups in a concentration dependent manner. Statistical analyses showed a significant difference between trabectedin treated cells compared to control (p<0.001).

Results showed that trabectedin exposure at increasing concentrations resulted in higher population of early apoptotic cells. Based on our data we conclude that trabectedin induces apoptotic cell death in all groups tested with a marked increase in early apoptosis at 48 hours. Trabectedin induced the total apoptotic cells in DU-145 cell line (Fig 3A), DU-145 CSCs (Fig 3B), DU-145 non-CSCs (Fig 3C), PC-3 cell line (Fig 3D), PC-3 CSCs (Fig 3E) and PC-3 non-CSCs (Fig 3F) in a dose-dependent manner.

### Caspase-3, caspase-8, caspase-9, p53 and bcl-2 modulate flavopiridol-associated apoptosis

**Fig 4:**
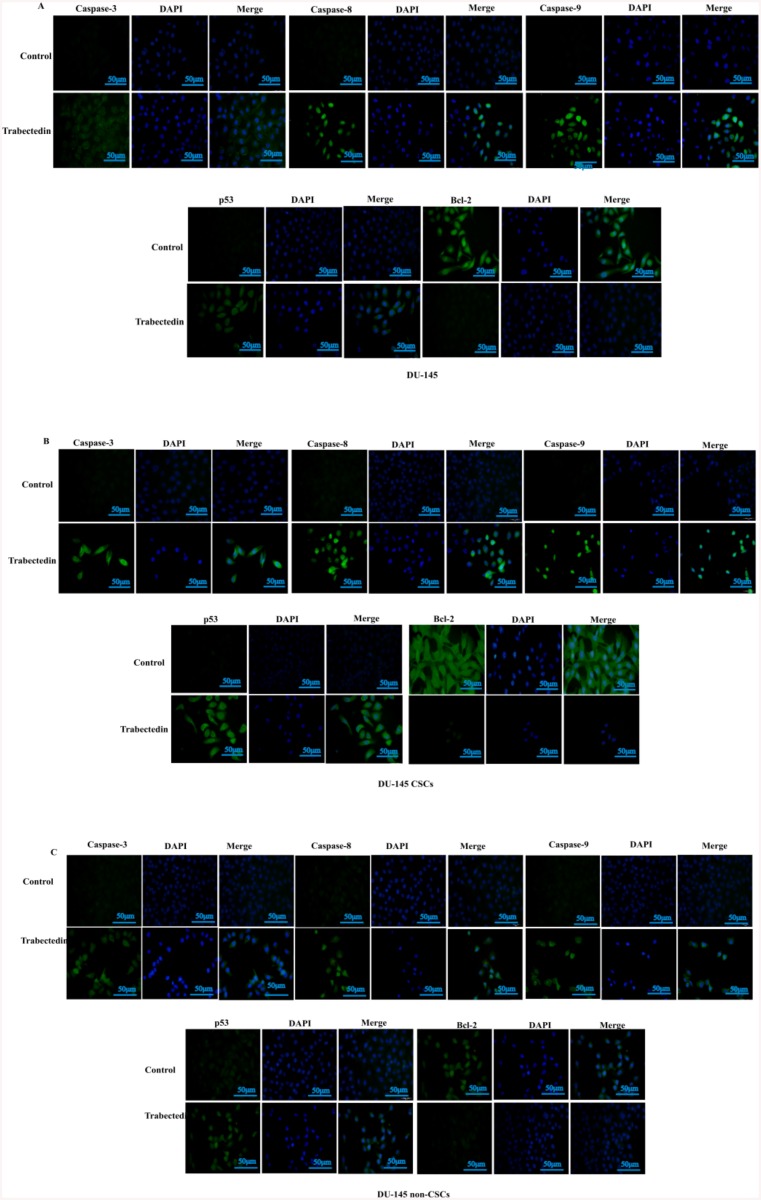
Immunofluorescence staining of caspase-3, caspase-8, caspase-9, p53 and bcl-2. (A) DU-145, (B) DU-145 CSCs, (C) DU-145 non-CSCs. Following treatment with 10 nM trabectedin; caspase-3, caspase-8, caspase-9, p53 and bcl-2 were visualized using FITC-conjugated secondary antibody (green). Nuclear staining was visualized using DAPI (blue) staining. Images are representative of three independent experiments. The scale bar stands for 50μm.

Immunofluorescence staining for caspase-3, caspase-8, caspase-9, p53 and bcl-2 supported our results and gave information on the involved apoptotic pathway. In DU-145 cell line (Fig 4A), DU-145 CSCs (Fig 4B) and DU-145 non-CSCs (Fig 4C) treated 10 nM trabectedin resulted in a significant increase in immunofluorescence staining of caspase-3, caspase-8, caspase-9 and p53. In contrast, immunofluorescence staining of bcl-2 was visibly decreased compared to the control. Similar observations were recorded in PC-3 cell line, PC-3 CSCs and PC-3 non-CSCs: immunofluorescence staining of caspase-3, caspase-8, and p53 were increased and immunofluorescence staining of bcl-2 was decreased. No significant changes were observed in immunofluorescence staining of caspase-9.

### Cell cycle regulation with high-dose trabectedin treatment

To assess whether Trabectedin-induced growth inhibition of cells is mediated via alterations in cell cycle, we examined the effect of trabectedin on cell cycle distribution by dead cell assay kit. Increase in incubation time resulted in an increase of the amount of cells in G2/M in all groups; particularly with high-dose (10 and 100 nM) treatments.

**Fig 5:**
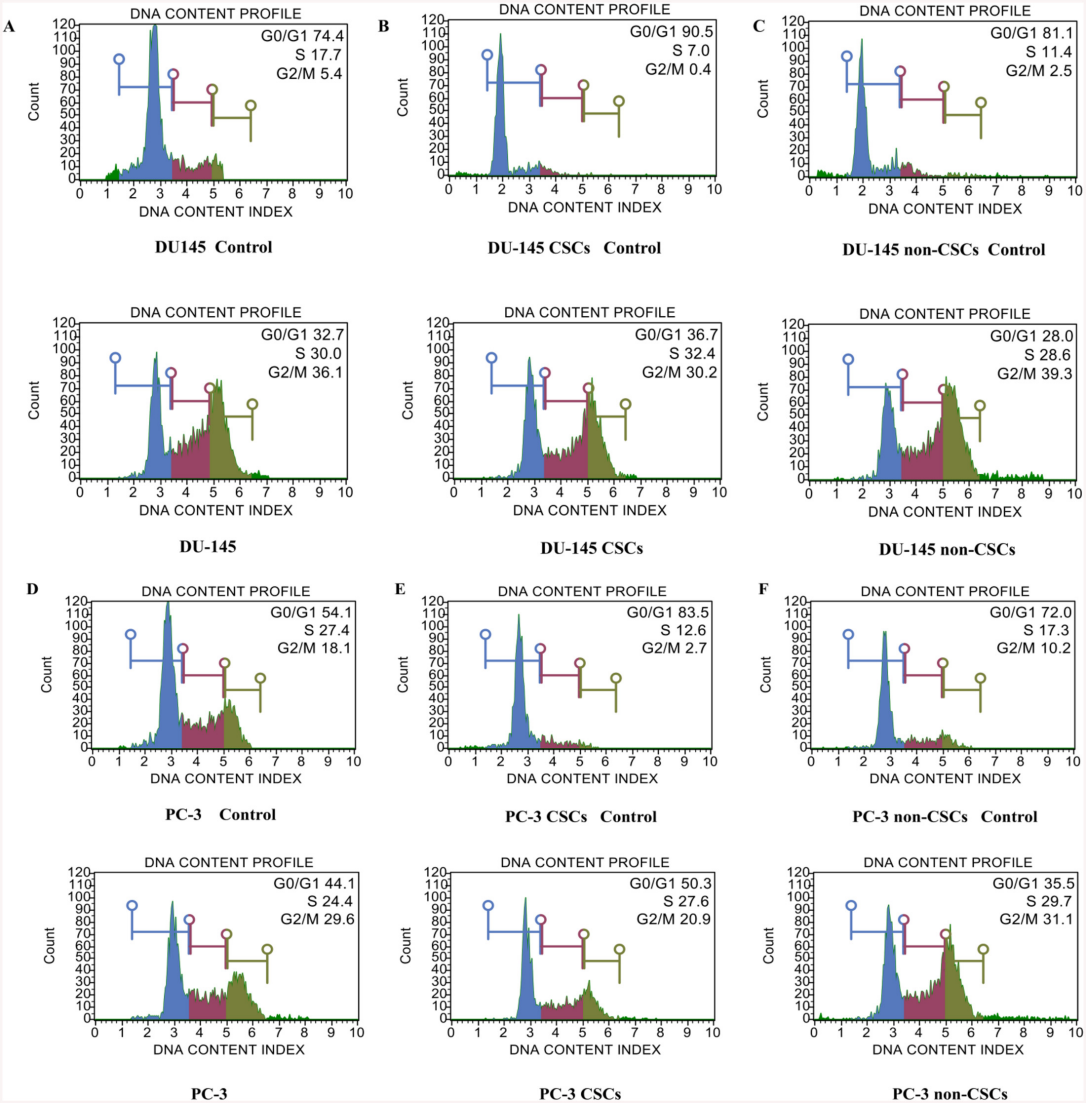
Cell cycle analysis obtained by treating with trabectedin according to the Muse™ cell cycle kit. (A) DU-145, (B) DU-145 CSCs, (C) DU-145 non-CSCs, (D) PC-3, (E) PC-3 non-CSCs, (F) PC-3 CSCs. The cells were treated with 10 nM trabectedin for 48 h. The percentage of cells in G0/G1, S and G2/M phases was then calculated using a Muse cell analyzer. Histograms from a representative experiment show the effect of trabectedin on cell cycle profile. Notably, trabectedin significantly influenced the cells in the G2/M phase, particularly in the high-dose treatment. Data shown here are from a representative experiment repeated three times with similar results.

After 48 h of 0.1 nM trabectedin treatment cell populations in the G0/G1, S and G2/M phases were 55.3, 27.6 and 17.1%, respectively in DU-145 cell line, and 58.3, 23.1 and 18.6%, respectively in DU-145 CSCs. Following 1 nM trabectedin incubation, the percentages were 54.0, 21.2 and 24.8%, respectively in DU-145 cells; and 50.1, 26.9 and 23.0%, respectively, in DU-145 CSCs. Incubation with 10 nM trabectedin resulted in the percentages of 35.7, 29.8 and 34.5% respectively in DU-145 cell line and 37.0, 32.5 and 30.4% respectively in DU-145 CSCs (Fig 5A and 5B). When cells were treated with 100 nM trabectedin, the percentages of the three classes of cells were 28.0, 29.5 and 42.5%, respectively, in DU-145 cell line; and 38.1, 21.8 and 40.0%, respectively in DU-145 CSCs. In DU-145 non-CSCs, cell populations in G0/G1, S and G2/M phases were 56.2, 24.4, 19.4% respectively, after 48 h incubation with 0.1 nM trabectedin. The percentages of the three classes of cells with 1 nM trabectedin incubation were 50.1, 19.3, 30,6%; with 10 nM trabectedin and 37.2, 22.9 and 39.9% (Fig 5C); with 100 nM trabectedin 25.5, 29.1, 45.4% respectively after 48 h incubation.

After 48 h of treatment with 0.1 nM trabectedin, cell populations in the G0/G1, S and G2/M phases were 57.0, 24.6 and 18.4% respectively in the PC-3 cell line, and 69.4, 12.7 and 17.8%, respectively in PC-3 non-CSCs. Incubation with 1 nM trabectedin, the percentages of the three classes of cells were 45.8, 25.5 and 28.6%, respectively in the PC-3 cell line and 48.7, 22.9 and 28.3%, respectively in PC-3 non-CSCs. Incubation with 10 nM trabectedin, the percentages of the three classes of cells were 49.5, 17.7 and 32.7% (Fig 5D), respectively in the PC-3 cell line and 35.8, 31.1 and 33.0% (Fig 5E), respectively in PC-3 non-CSCs. Incubation with 100 nM trabectedin, the percentages were 43.8, 18.9 and 37.3%, respectively in the PC-3 cell line and 28.0, 26.4 and 45.6% respectively in PC-3 non-CSCs. Similar observations were recorded in PC-3 CSCs. Exposure of these cells to trabectedin concentrations of 0.1, 1, 10 and 100 nM resulted in 76.4, 62.7, 50.3, 45.8% arrest in the G0/G1 phase, respectively; and 15.0, 22.0, 27.6, 26.3% arrest (respectively) in the S phase, and 8.6, 15.3, 20.9, 27.9% arrest (respectively) in the G2/M phase (Fig 5F). These results indicated that the growth inhibition observed in all groups treated by trabectedin is mainly associated with G2/M phase arrest.

### Comparison of spheroid forming ability of CSCs and non-CSCs

**Fig 6:**
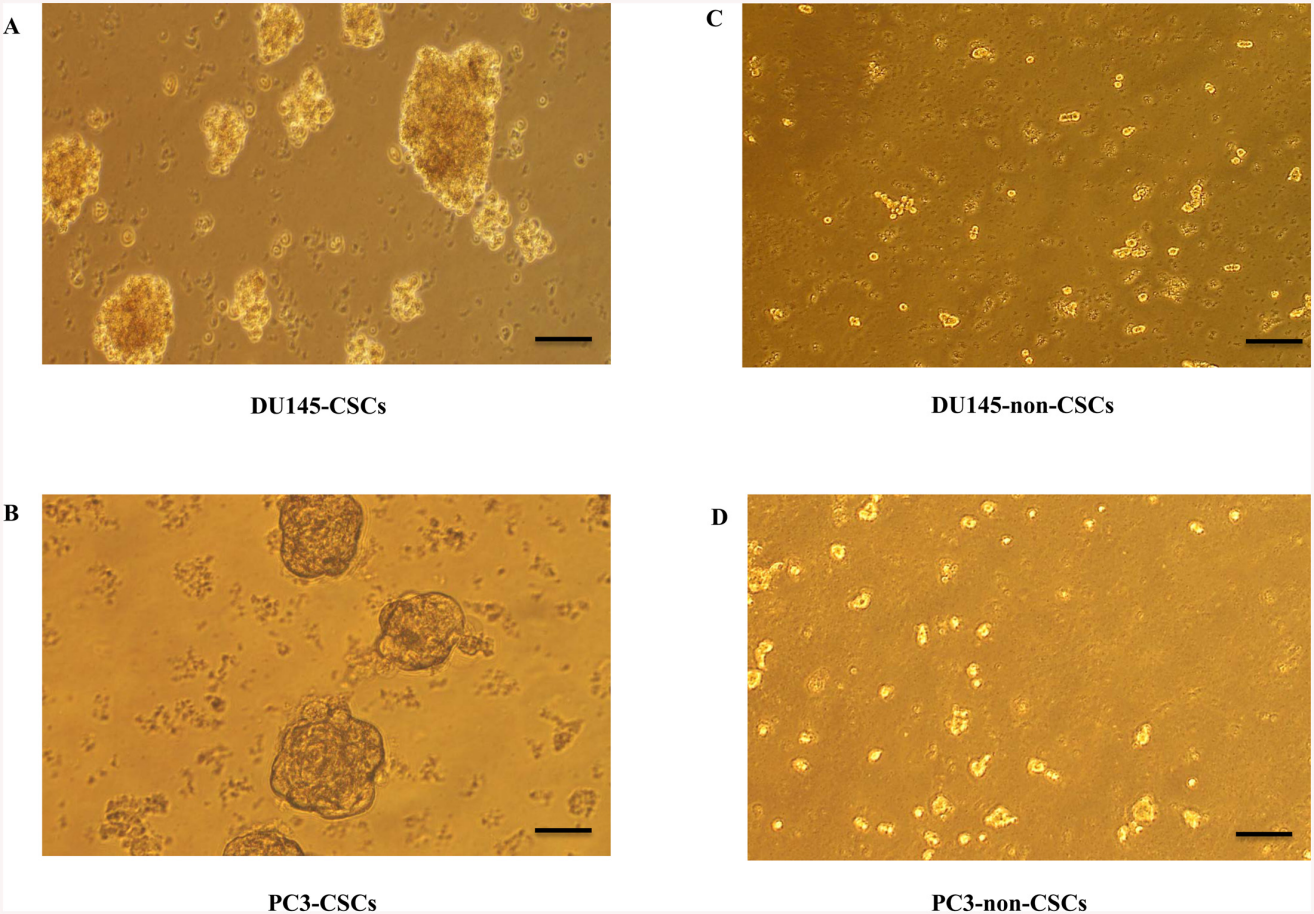
The spheroid formation potential of CSCs and non-CSCs. A) DU-145 CSCs, B) PC-3 CSCs, (C) DU-145 non-CSCs, (D) PC-3 non-CSCs. CD133^high^/CD44^high^ human prostate CSCs were able to form spheroids; whereas non-CSCs failed.

DU-145 and PC-3 CSCs and non-CSCs were grown in 3D non-adherent culture conditions. Spheroid formation was evaluated under a phase contrast microscope. CD133^high^/CD44^high^ human prostate CSCs (Fig 6A and 6B) were able to form spheroids; whereas non-CSCs failed (Fig 6C and 6D).

### Inhibition of sphere formation with trabectedin

**Fig 7:**
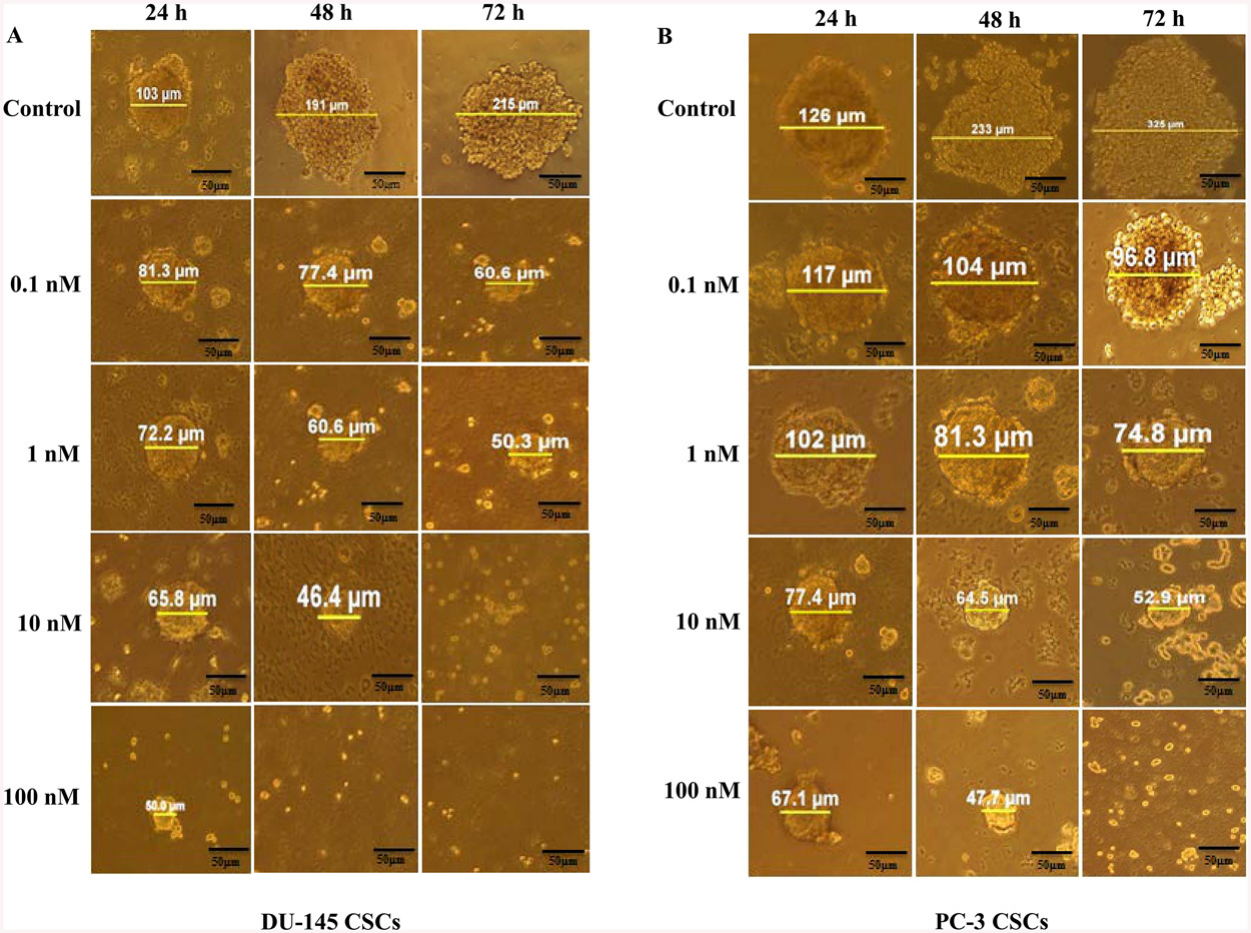
Effect of trabectedin on the spheroids in CD133^high^/CD44^high^ DU-145 and PC-3 CSCs. (A) DU-145 CSCs, (B) PC-3 CSCs. Ability of sphere formation of CD133^high^/CD44^high^ DU-145 and PC-3CSCs is markedly suppressed in a dose-dependent manner from the beginning of the spheroid constitution. After the beginning of the multicellular tumor spheroid formation, trabectedin was added at various concentrations of trabectedin (0.1, 1, 10, 100 nM) for 24, 48 and 72 h. A significant decrease was observed in the diameter of spheroid formation. The results are representative of data collected from at least three experiments.

**Fig 8:**
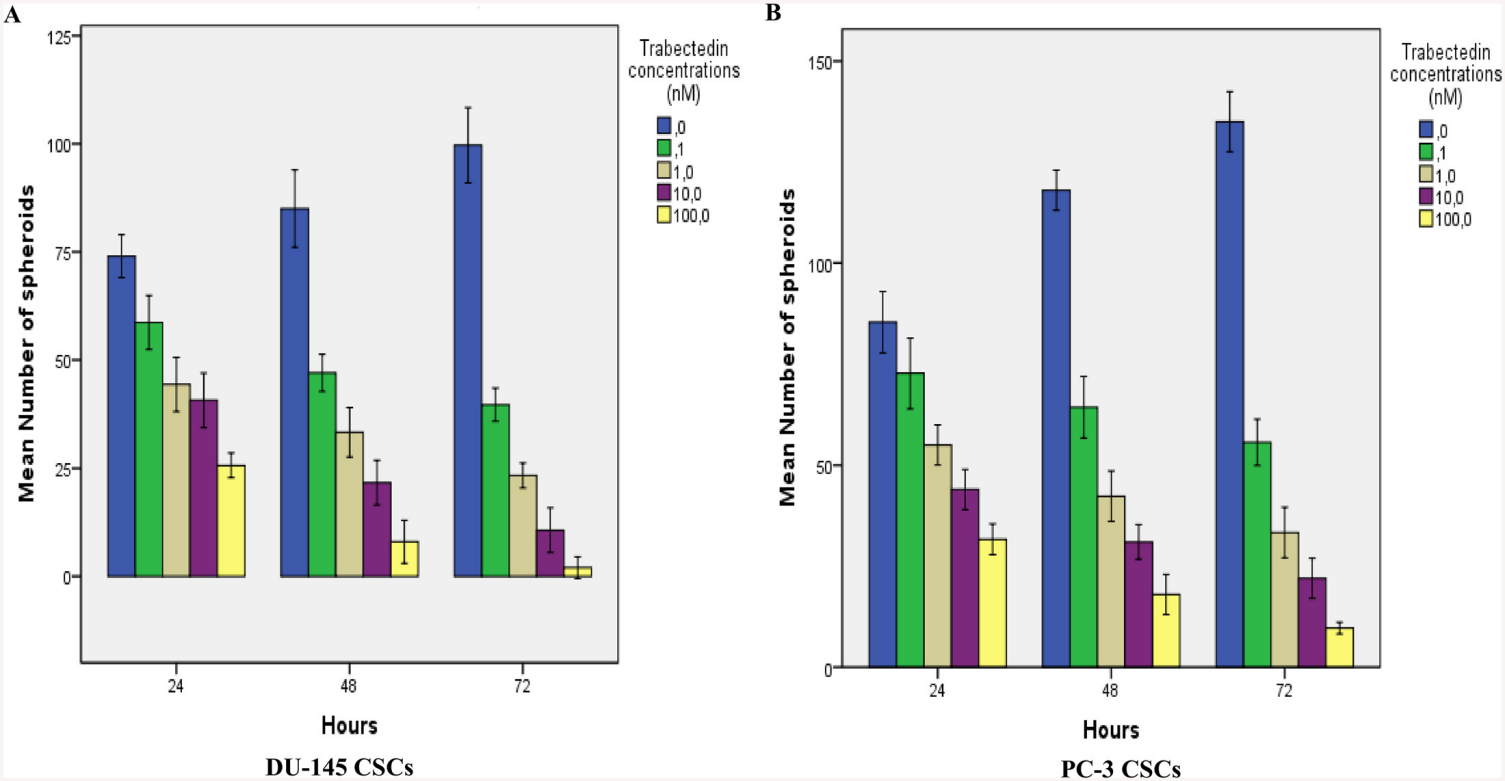
Effect of trabectedin on number of spheroids in CD133^high^/CD44^high^ DU-145 and PC-3CSCs. (A) DU-145 CSCs, (B) PC-3 CSCs. After the beginning of the multicellular tumor spheroid formation, trabectedin was added at various concentrations of trabectedin (0.1, 1, 10, 100 nM) for 24, 48 and 72 h. The number of spheroids of 15–20 random fields were counted and calculated. Trabectedin reduced the number of spheroids of DU145 CSCs and PC3 CSCs in a dose and time dependent manner.

DU-145 CSCs and PC-3 CSCs formed tumoroids at day five and three, respectively. The early spheroids were incubated for 24, 48 and 72 h and cells were treated with 0.1, 1, 10 and 100nM trabectedin. The number and diameter of colonies within each well was determined each day under the microscope. Measurements of tumoroid size and numbers revealed a dose-dependent cytotoxicity in treated tumoroids when compared to untreated controls. Trabectedin reduced the number and diameter of spheroids of DU145 CSCs and PC3 CSCs in a dose and time dependent manner (Figs 7 and 8). With increasing doses of trabectedin, increased cell death was observed to accompany the dissolution of spheroids of DU145 CSCs and PC3 CSCs.

### E-cadherin expression decreased in CD133^high^/CD44^high^ prostate CSCs with trabectedin

**Fig 9:**
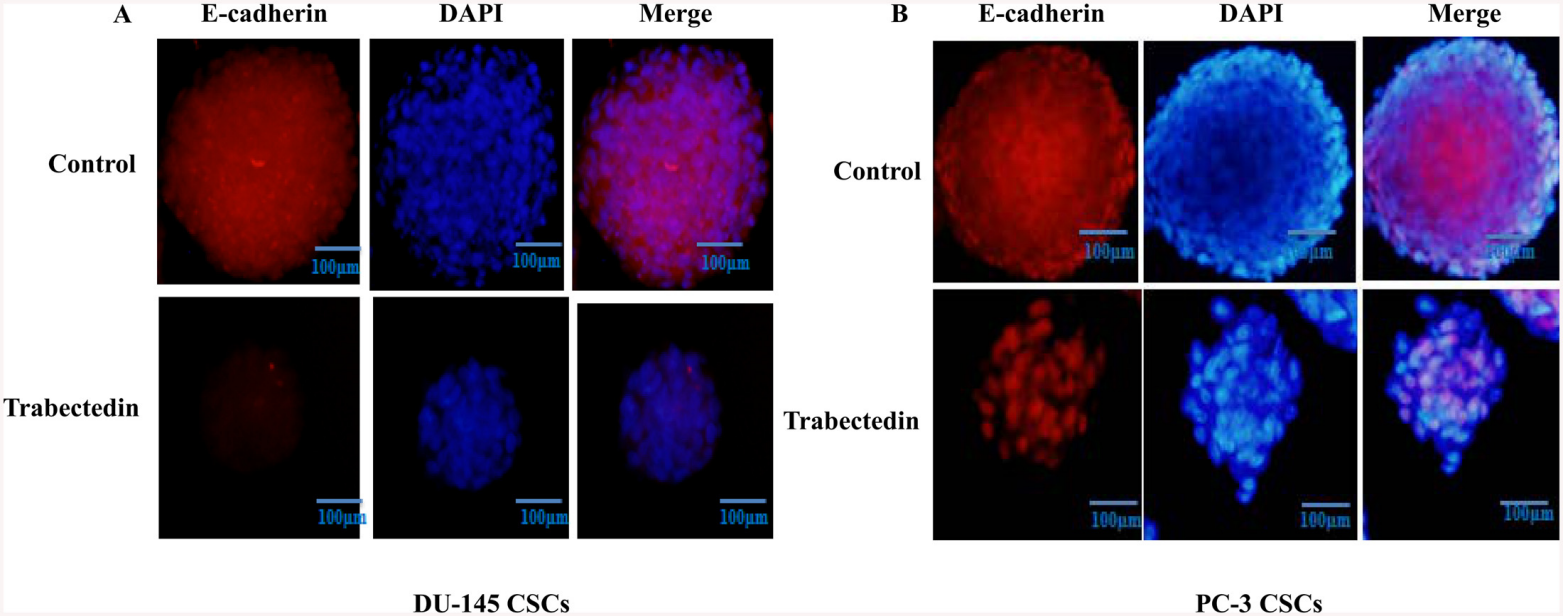
E-cadherin expression in DU145 and PC-3 CSCs spheroids. (A) DU-145 CSCs, (B) PC-3 CSCs. After the multicellular tumor spheroid formation, DU-145 CSCs and PC-3 CSCs spheroids were treated with trabectedin. Trabectedin disrupted E-cadherin-mediated cell-cell interactions in multicellular spheroids of DU-145 and PC-3 CSCs. E-cadherin were visualized using Alexa fluor ^®^ 594 -conjugated secondary antibody (red) Nuclei are stained with DAPI (blue). Scale bar: 100 μm.

To investigate the role of trabectedin in the expression of E-cadherin-mediated cell-cell adhesion in DU-145 and PC-3 CSCs grown under three dimensional conditions in vitro, we examined the immunofluorescence staining of E-cadherin. Our immunofluorescence assays results showed that after treatment of DU-145 and PC-3 CSCs with IC_50_ doses trabectedin, E-cadherin expression significantly decreased compared to control (Fig 9).

## Discussion

Our study is the first to demonstrate the cancer preventive effects of trabectedin on human prostate CSCs. Recent cancer studies have revealed that a minority population of cells in tumors bulks is responsible for tumor initiation, growth and resistance to conventional treatments. Most of cancer drugs under investigation, while killing the bulk of tumor cells, ultimately fail to eleminate CSCs, which cause of tumor recurrence and metastasis. Therefore, attention has been focused on defining new anticancer drug for cancer prevention and therapy by eliminating CSCs.

Trabectedin has the potential to be an effective chemotherapeutic agent for the development of novel treatment strategies that specifically target prostate CSCs. Our data strongly suggest that trabectedin inhibits the growth of prostate CSCs in a dose-dependent manner, via cell cycle arrest and apoptosis. Trabectedin exhibits a strong activity against a variety of tumor cell lines of different origins *in vitro* and *in vivo* models. Clinical studies with trabectedin has demonstrated its antineoplastic activity against various human tumors including soft tissue sarcoma and ovarian cancer [15, 16]. Our study is the first study investigating the effects of trabectedin on prostate cancer stem cells and will be beneficial for future developments of novel treatment strategies for prostate cancer.

Human prostate cancer cell lines derived from bone metastasis (PC-3) and brain metastasis (DU-145) are widely used for in vitro prostate cancer research studies and proved to be a powerful tool for the discovery of new anticancer drugs and for understanding the molecular mechanisms involved in cell resistance to chemotherapeutics already used in the treatment of cancer [17]. Investigation of different cell lines may provide a valuable means for preliminary assessment of new therapeutic agents.

Cytotoxic and apoptotic effects of trabectedin has been shown previously in various cancer cell types including leukemia [14], breast cancer [18] and lung adenocarcinoma [19]. However the effect of trabectedin on cancer stem cells is still a matter of debate. There is no data in the literature on the effects of trabectedin on CSCs *in vivo* or *in vitro*. Research revealed that CSCs are phenotypically and functionally different from non-CSCs. CSCs are more resistant to radiotherapy and chemotherapy compared to the bulk of tumor cells (non-CSCs). In our study, we examined the cytotoxic effects of trabectedin on human prostate CSCs and non-CSCs. Our data showed that non-CSCs are more sensitive to trabectedin when compared to CSCs. We also showed that trabectedin induces cytotoxicity in prostate CSCs in a dose- and time-dependent manner; whereas it is less effective (cytotoxic effects) against the non-tumour cell line RWPE-1. Cytotoxic anticancer drugs kill cells with different mechanisms of action; such as microtubule instability, inducing DNA damage and apoptosis in tumor cells. Our results suggest that trabectedin is cytotoxic to prostate CSCs in vitro, by causing a cell cycle arrest at G2/M and inducing apoptosis. The mechanistical action of trabectedin on the cell cycle is dependent on its binding to DNA and eventually creating DNA double-strand breaks that alter the normal function of DNA repair and transcription processes resulting in cell cycle arrest. DNA structure gives rise to two well-defined clefts known as the major and minor grooves [11] both being sites of attack for many antitumor drugs. Many anti-tumor drugs such as cisplatin bind to the DNA major groove and lead to cell cycle arrest and cell death. On the other hand, other antitumor drugs such as trabectedin, lurbinectedin and mitomycin C bind to the minor groove. Trabectedin reacts with certain guanines in the minor groove of DNA, forming a covalent bond that gives rise to DNA double-strand breaks that triggers a cascade of events that ultimately leads in G2-M cell cycle arrest and apoptosis [11]. The selectivity of trabectedin also seems to be a great advantage for its use as a therapeutic agent against prostate cancer.

Most of the chemotherapeutic drugs in current use are expected to inhibit the growth of cancer cells [20]. Understanding the mechanisms involved in the process of trabectedin-mediated apoptosis in prostate CSCs is important will provide valuable information for the development of novel treatment strategies. We found that trabectedin induced apoptosis in DU-145 and PC-3 prostate CSCs in a dose-dependent manner, suggesting that trabectedin-mediated cell growth inhibition can be related to the induction of apoptosis. Preusser et al. [21] demonstrated that trabectedin induces both early and late-stage apoptosis in human meningioma cells. Other research groups have suggested that trabectedin induces apoptosis through extrinsic and/or intrinsic pathways [14, 19, 22]. Our data also shows that trabectedin-induced growth inhibition and apoptosis is associated with increased expression of caspase-3, caspase-8, caspase-9, p53; and decreased expression of bcl-2 in CD133^+high^/CD44^+high^ prostrate CSCs. Atmaca *et al*. demonstrated that the levels of caspases, Bad and Bax increased and the levels of Bcl-2 and Bcl-xl decreased in breast cancer.

The quiescence of cancer stem cells has critical biologic importance in maintaining the chemotherapy resistant population. The protection of the quiescent state depends on transcription factors, signaling proteins, cell cycle proteins. Therefore, targeting cell cycle regulatory mechanisms of quiescent cancer cells will be new perspectives for the elimination of CSCs. Recently, many chemotherapeutic agents have been shown to impart anti-proliferative effects by arresting cell division at certain checkpoints in the cell cycle. In this study, we observed that high doses of trabectedin induce G2/M phase arrest in prostate CSCs. Previous reports have shown that antiproliferative activity of trabectedin is caused by inducing an accumulation of cells in S phase, eventually resulting in a G2/M block [12]. It has been shown that aside from interfering with the cell cycle by causing a G2/M block, trabectedin also affects cell-cycle regulator genes and genes involved DNA repair pathways [11, 12, 13].

Our data suggested that CSCs and non-CSCs have different sensitivity to trabectedin. Our results show that CSCs are less sensitive to trabectedin than CD133^low^/CD44^low^ cells. Recent work on trabectedin, and CSCs metabolism have both contributed to our understanding of the different modes of action trabectedin displays in CSCs and non-CSCs. One possible explanation is that CD133^high^/CD44^high^ prostate CSCs have an increased DNA damage checkpoint activation and damage repair mechanisms relative to non-CSCs. The repair of DNA double-strand breaks induced by trabectedin is more rapid and effective in CSCs compared with non-CSCs. The second possible explanation relies on the quiescent state observed in CSCs. CSCs are a slow cycling cell population maintained in the G0 phase of cell cycle (quiescent state). The quiescent state protects cancer stem cells from cell division associated damage. Another possible explanation is that the CSC subpopulation is more resistant to trabectedin-induced apoptosis than non-CSCs.

Recent studies have demonstrated that in vitro 3D tumor cell culture model mimic features of the in vivo microenvironmen better than simple two-dimensional monolayers. This provides a better opportunity to understand drug resistance mechanisms of cancer cells [23]. The ability to form spheres has been established as one of the characteristic features of CSCs, and spheroid formation assay is often used for drug screening. In this study, CD133^+high^/CD44^+high^ DU-145 and PC-3 CSCs were able to form spheroids in non-adherent culture, suggesting the presence of cancer stemlike cells within these cells. Our results showed a strong inhibitory effect of trabectedin on the sphere-formation ability interpreted by a decrease in sphere size and number of cells per sphere. Cell adhesion systems play a crucial roles in a wide variety of cellular and developmental processes including cell migration, morphology, differentiation and proliferation. E-cadherin, the major adhesion molecule mediating tight cell-cell interaction, is known as a mediator of tumor spheroid formation in prostate cancer [8]. Our data reveals that CD133^+high^/CD44^+high^ DU-145 and PC-3 CSCs has ability to form spheroids correlating with E-cadherin expression. E-cadherin dependent sphere formation has also studied in human tumor cell lines, in which it has been demonstrated that E-cadherin-mediated cell-cell interactions form compact spheroids [9, 24, 25]. Vieweg et al. demonstrated that only E-cadherin positive (E-cad^+^) cell subpopulations expressed high levels of the embryonic stem cell markers (SOX2, OCT3/4, Nanog, Klf4 and c-Myc), formed colonies, and grew as spheres under non-adherent conditions in prostate cancer [26].

## Conclusions

This is the first *in vitro* study showing that trabectedin has profound activity against prostate CSCs. Our collective data suggest that trabectedin inhibits cell growth and spheroid formation of prostate CSCs through the induction of cell cycle arrest and apoptosis. Trabectedin induces apoptosis by up-regulation of caspase-3, caspase-8, caspase-9, p53 and down-regulating pro-survival molecules such as bcl-2. These findings indicate that trabectedin may have a potential therapeutic value against prostate CSCs. However further research should investigate whether targeting CSCs with trabectedin could be of clinical benefit in an appropriate in vivo model.
